# Propagation of Bankruptcy Risk over Scale-Free Economic Networks

**DOI:** 10.3390/e24121713

**Published:** 2022-11-24

**Authors:** Joseph Andria, Giacomo di Tollo, Jaan Kalda

**Affiliations:** 1 Dipartimento di Scienze Economiche, Aziendali e Statistiche, Università degli Studi di Palermo, 90100 Palermo, Italy; 2 Dipartimento di Diritto, Economia, Management e Metodi Quantitativi, Università degli Studi del Sannio, 82100 Benevento, Italy; 3 Department of Cybernetics, Tallin University of Technology, 10115 Tallin, Estonia

**Keywords:** financial contagion, bankruptcy risk, scale-free networks, targeted attacks, Shannon entropy

## Abstract

The propagation of bankruptcy-induced shocks across domestic and global economies is sometimes very dramatic; this phenomenon can be modelled as a dynamical process in economic networks. Economic networks are usually scale-free, and scale-free networks are known to be vulnerable with respect to targeted attacks, i.e., attacks directed towards the biggest nodes of the network. Here we address the following question: to what extent does the scale-free nature of economic networks and the vulnerability of the biggest nodes affect the propagation of economic shocks? We model the dynamics of bankruptcies as the propagation of financial contagion across the banking sector over a scale-free network of banks, and perform Monte-Carlo simulations based on synthetic networks. In addition, we analyze the public data regarding the bankruptcy of US banks from the Federal Deposit Insurance Corporation. The dynamics of the shock propagation is characterized in terms of the Bank Failures Diffusion Index, i.e., the average number of new bankruptcies triggered by the bankruptcy of a single bank, and in terms of the Shannon entropy of the whole network. The simulation results are in-line with the empirical findings, and indicate the important role of the biggest banks in the dynamics of economic shocks.

## 1. Introduction

In today’s context of global financial interconnectedness, risk reduction offered by country diversification may have its counterpart in the widespread diffusion of a contagion from countries experiencing severe local economic crises [1,2,3,4]. A small, even a single shock from a country may spread to other economically connected countries, thus causing severe implications to the whole system. As a result, the behaviour of how the initial shock propagates across the system may be very different depending on the countries’ network structure, which plays a fundamental role in propagation dynamics.

In this framework, it is important to model the dynamics of the *contagion*, referring to financial risk in general, with an emphasis on bankruptcy risk: to this end, many approaches have been suggested by the literature (see Section 2 for a broad overview). In this direction, the contagion could be modelled as a spreading process over a complex network, in which the structure of the network itself has a measurable impact on the whole spreading process.

There is evidence that economic networks exhibit a *scale-free* structure, cf. [5,6,7,8,9,10]. Scale-free networks are networks in which the nodes degree distribution follows a power law, and one may notice the presence of large hubs (the degree of a node is defined as the number of links connected to this node).

Based on the analogy between the bankruptcy risk spreading over financial networks, and a virus spreading over a network of human contacts, we can make use of research results regarding virus spreading processes. We shall also borrow terminology from epidemiology, e.g., the concept of superspreaders—the nodes of the network with a large number of links which can transmit a contagion to many other nodes once infected, and the concept of the reproduction number $R_{E}$—the number of nodes infected by a single node on average. In the context of bankruptcies, however, we’ll be referring to $R_{E}$ as *the Bank Failures Diffusion Index*. It has been shown [11] that the value of the effective reproduction number $R_{E}$ is dominated by the superspreaders. For a scale-free network, the degree of the largest nodes depends on the size of the network. Hence, the value of $R_{E}$ grows with the size of the network. Furthermore, it has been also shown, in [11], that if a node catching the contagion results in the node being removed from the network, the disruption of the network starts from the biggest nodes. This phenomenon has been referred to as *self-targeting attacks* due to the fact that the biggest, and, hence, the most vulnerable nodes are being targeted automatically. The self-targeting happens because the nodes with the highest number of connections have the highest likelihood of getting *infected*. As a result, the value of $R_{E}$ starts to rapidly decrease over time.

In this paper, we aim to test whether a financial contagion process can be modelled by means of a scale-free network structure. To this extent, we analyse publicly accessible data from the Federal Deposit Insurance Corporation and we investigate to what degree the scale-free nature of the economic network and the vulnerability of the biggest nodes affect the propagation of economic shocks over the network itself. Using the aforementioned scale-free network approach, we address the dynamics of financial contagion across the banking sector.

The paper is structured as follows: Section 2 outlines the related literature on the topic; Section 3 outlines the methodology of our contribution, while the proposed scale-free model of the financial contagion/diffusion is presented in Section 4. Results are provided in Section 5, before we conclude our contribution in Section 6.

## 2. Related Work

The plethora of contributions about financial contagions have improved knowledge of this phenomenon, by outlining the importance of extreme negative outcomes and the phenomenon of increased interdependence. Several methods have been proposed to model or explain the contagion: GARCH [12], standard quantile and Bayesian quantile regression [13], non-linear Markov-switching model [14], agent-based models [15], copulas [16], and the canonical model [17], just to name a few. A unified framework to identify the channels for the international transmission of financial shocks has been provided by [18], while other contributions investigate the contagion mechanism in different sectors [19], and countries: in this context, most contributions agree on the fact that macro-prudential authorities have to adopt a pan-European perspective [20]. In addition, nation-wide assessments have been performed [21] showing that, say, the risk of cross-border bank contagion in the European Union increased during 1990s.

Providing an extensive survey of the literature about contagions is out of the scope of the current contribution, and we forward the interested reader to [22,23] for an overview. Instead, we want to stress that several contributions introduce network analysis to model the contagion: Ref. [24] introduces networks with both directed and undirected links, finding that stable networks can be asymmetric and connected, being able to capture the main features of inter-industry and financial networks. The authors of [25] introduce network analysis to identify the connections between risk and sentiment contagion; the authors of [26] investigate the *tails* of the phenomenon; while [27] builds a multiple network of the bank system by coupling the bank system network with the depositor’s social network in order to study the interaction and impact mechanism due to the contagion of bank risk combined with panic sentiments of depositors; the authors of [28] adopt a complex network approach to assess the level of heterogeneity and cohesiveness among firms that make use of minibonds as a source of financing.

Although scale-free networks have been theoretically investigated to model contagion effects [29], their application to the analysis of bankruptcy risk is still limited: Ref. [30] introduces a comparative analysis, Ref. [31] performs a computer-based simulation, and Ref. [32] investigates the European scenario, on top of some contributions tailored to specific sectors, such as re-insurance [33], real estate [34], and portfolio diversification [35]. Our contribution is intended to fill this gap, by applying scale-free networks to modelling the effects of bankruptcy outbreaks across the banking sector.

Moreover, our approach can prove to be an effective aid to examining the distress spillover effects across industries, such as the propagation of firm-level idiosyncratic shocks in production networks. In particular, [36] shows that in a competing network of industries, a distress shock can propagate to other industries through common *major* players and its (indirect) spillover effects can deteriorate the profit margins of unaffected industry peers. Ref. [37] identifies idiosyncratic shocks with the occurrence of natural disasters and finds that affected suppliers impose substantial output losses on their customers, especially when they produce specific inputs. These losses may cause adverse implications, such as significant market-value losses which, in turn, spillover to other suppliers. In [38], an alternative model of network formation is proposed that parsimoniously incorporates realistic features of US firms’ buyer–supplier relationship. The model accounts for processes of vertex (firm) death and the restoration of those edges (buyer–supplier relationships) between surviving firms. New edges are also allowed to be formed, either by considering a scale-free network structure (new edges are more likely to be attached to nodes with a high degree) or randomly.

## 3. Methodology

In order to verify if financial contagion spreads in real world like a scale-free network or not, we used publicly accessible data from the Federal Deposit Insurance Corporation (FDIC) website. The FDIC is an independent agency created by Congress to maintain stability and public confidence in the US nation’s financial system. To accomplish this mission, the FDIC insures deposits; examines and supervises financial institutions for safety, soundness, and consumer protection; makes large and complex financial institutions resolvable; and manages receiverships. It is also appointed as a receiver for failed banks and keeps track of a neat failed-bank list, where 563 bank failures are recorded in the US since 1 October 2000 until October 2020 (see Data Availability Statement).

**Figure 1:**
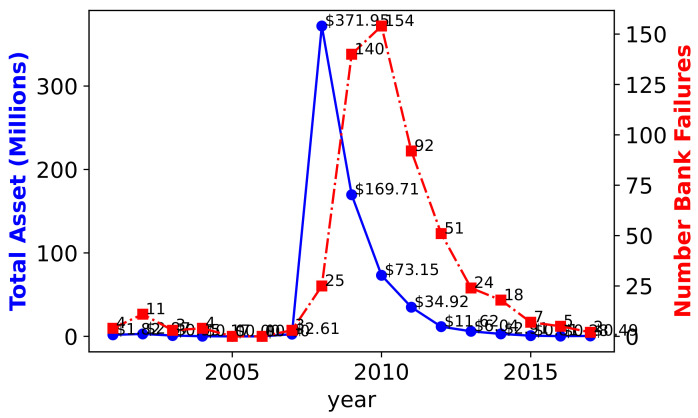
A snapshot of the data used in the study (source: The Federal Deposit Insurance Corporation (FDIC).

In Figure 1, we show a snapshot of the available data displaying the yearly number of bank failures along with their approximate total asset volume (in USD ). The bank name, the city, the closing date and the approximate deposit US dollar volume are also available along with the above listed data.

**Figure 2:**
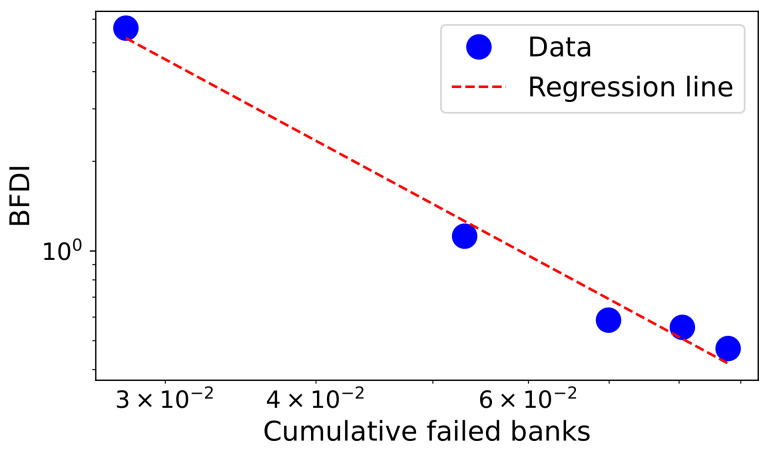
The yearly observed Bank Failures Diffusion Index (BFDI) against the cumulative number of failed banks *f* (Period: 2009–2013, Source: FDIC). The red dashed line depicts a power law fit such as BFDI $\propto f^{-\alpha }$ with α≈2.

In Figure 2, we plotted in a log–log scale the yearly Bank Failures Diffusion Index (BFDI) for the period 2009–2013 against the cumulative number of failed banks. The reported BFDI is calculated as the ratio between the number of new end-of-year failures and the ones observed one period ahead, while the cumulative number of failed banks *f* is averaged over the total number of (commercial) banks at each investigated year. For maximum data stability, and following the methodology applied in Ref. [11], we consider the period from 2009 to 2013, as we assume that the 2008 financial global crisis reveals its full extent from 2009 and it starts to largely fade out by the end of 2013.

The red straight dashed line in the figure depicts a power law fit of the US banks’ Failures Diffusion Index as a function of the cumulative number of failed banks, which yields an exponent α≈2. This evidence would suggest that it might be reasonable to model the observed diffusion behaviour as a *scale-free network*. In other words, what we want to investigate is if the depicted behaviour in Figure 2 is, effectively, the consequence of the scale-free nature of the banks’ failure-spreading process.

As a matter of fact, the scale-free nature assumption of the failure-spreading process, if verified, would have significant implications, in terms of the understanding of the network dynamics, and for implementing optimal control strategies over the network itself. For example, as it is well-known in the literature, scale-free networks are highly sensitive to so-called *targeted attacks*, which aim to compromise network integrity through disrupting the biggest nodes [39,40,41]. If, as hypothesized, a *self-targeting-attacks* scenario is playing out in the network of inter-related companies, the biggest nodes, i.e., the nodes with the highest degree, become “destroyed” at the very early stages of the bankruptcy spreading throughout the network. This is a strong possibility because the probability of a node being contaminated by a malicious event is proportional to the degree of the node itself. This has both crucial and practical implications in averting bankruptcy diffusion and preventing a deep cross-border or cross-sectoral financial contagion. The identification of the likely “infectious” superspreaders within the network by economic and financial authorities would ensure better decision making and policy implementation, such as dedicated financial support or special monitoring actions.

## 4. The Scale-Free Model of Financial Contagion Spreading

Based on the considerations reported in Section 3 on the implication of the scale-free nature of financial contagion spreading across a network, we developed the following mathematical model:

(a) the network links are kept fixed along time with time-varying strength. The probability *p* of the diffusion of financial distress from a failed bank (node of the network) to a susceptible one is initially kept constant and, subsequently, we let it depend also on the (susceptible) node’s neighborhood structure. The rationale for this is that the financial distress of a defaultable node can be induced by a failed directly connected node, but it can be also triggered by the defaults of its neighbouring nodes. In a sense, we are arguing that (probably) the default of a big connected node has a much wider impact than that of a small one.
  In more detail, let $k_{i}$ be the degree of the *i*-th node, then the probability of a default of the *i*-th node at a certain time step is given as:
  (1) $$p_{i}=q_{i}+a_{i}\times \sum_{j}k_{j}^{b}$$
  where the sum is taken over all the infected nodes (of index *j*) connected to the *i*-th node, with $q_{i}=\frac{Q}{k_{i}^{g}}$ and $a_{i}=\frac{c}{k_{i}^{d}}$, with both *g* and *d* taking values in the real interval $\left[0,1\right]$. This equation can be interpreted as follows. The term $q_{i}$ describes the probability of a spontaneous default. We expect smaller companies to be more volatile; hence, we assumed that the spontaneous default probability is a decreasing power law of the node’s degree *k*, with the constant *Q* and the exponent *d* being model parameters. The second term describes the default triggered by the defaults of the connected nodes; we expect big infected neighbours to have a stronger impact, hence the exponent b>0. In addition, we expect big nodes to be less sensitive to the defaults of small neighbours, so the exponent d>0. One can conclude that the exponents *b* and *d* must be equal by arguing that if a certain node of size *k* is linked to an infected node of equal size *k*, the probability of a triggered default should be independent of the node size *k*. To sum up, we have two more model parameters, the constant *c* and the exponent *b*, while d=b.
(b) At the simulation start, all the network nodes are susceptible to falling into a default status which is induced into the network by selecting a random node. The simulation run is deemed successful if at least a threshold fraction of 5% of the network nodes fall into default.
(c) The period between the occurrence of financial distress and a subsequent default status is taken to be equal to one time step, which corresponds to an iteration, and each distressed node remains *active* for one time step. This means that if a node falls into distress at $t_{n}$, it will default at time $t_{n+1}$ and will neither be susceptible to default again nor induce a bankruptcy risk for any $t\geq t_{n+2}$. Here, the idea is that when a bank goes into bankruptcy, it is immediately rescued by ad-hoc financial support policies implemented either by national or international governance bodies;
(d) The networks we use are characterized by the cumulative degree distribution exponent κ, i.e., the number of nodes *n* with degree exceeding *k* scales as $n\approx k^{-\kappa }$;
(e) We also introduce the *Shannon Entropy* (SE) in order to quantify the complexity of the effective remaining network of healthy nodes with a single number. The SE is a concept introduced by [42] and its applications in information theory as well as in other scientific disciplines are now countless. Entropy is a measure of the randomness in a system: the noisier a system is, the less predictable it becomes and the higher entropy it has. In our case, it reflects the diversity of the nodes.
  We define the *entropy* of the network as $S=-\sum_{k}\left[P_{k}\times \ln\left(P_{k}\right)\right]$, where $P_{k}=\frac{N_{k}}{N}$, $N_{k}$ denotes the number of nodes of degree *k* and *N* the total number of nodes in that network. We consider how entropy evolves under different scenarios. The way entropy changes over time basically reflects how many big nodes failed and were “removed” from the network. We expect it to start decreasing during the contagion spreading, as the network is initialized with the full spectrum of node sizes and, as the big nodes become unhealthy and enter into a financial distress/bankruptcy status, the system becomes distributed over a smaller number of states (i.e., nodes of different sizes). Hence, decreasing entropy would imply that self-targeting attacks have been successful in removing big infectious nodes (banks).

## 5. Results

Simulations were run by means of the Python NetworkX package [43] using the scale_free_graph function, which returns a scale-free directed graph [44]. The above library function basically depends on the following four parameters: *N* (the number of nodes in the graph) α, β and γ, such that α+β+γ=1. The way these parameters are set has a direct impact on the cumulative degree distribution exponent of the *in* and *out* connections. We then converted the directed graph into an undirected one, avoiding self connections or double connections between nodes.

**Figure 3:**
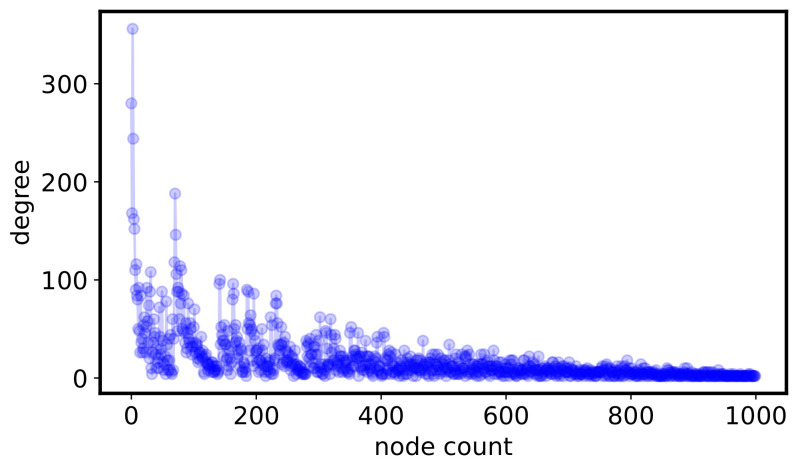
The node degree distribution of a simulated network by NetworkX.

For reproducibility, we report in Table 1 the parameters we used for performing the simulations while a node degree distribution obtained by a simulated run is depicted in Figure 3. In particular, the choice of parameters α=0.1, β=0.8 and γ=0.1 allows to obtain a power-law scaling exponent equal to κ=2.0, which is in-line with the observed pattern displayed in Figure 2.

**Figure 4:**
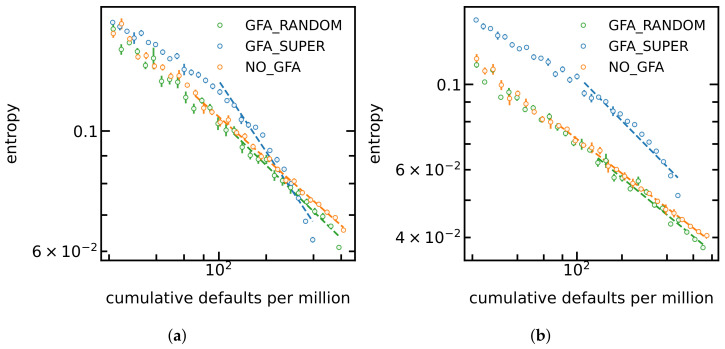
Entropy value versus the percentage of corrupted banks for different settings of the default-diffusion probability parameters and for different implementations of financial support policies (GFA: government financial aid, No_GFA: no financial aid is induced into the network, GFA_SUPER: financial aid is provided only to superspreaders, GFA_RANDOM: financial aid is induced randomly into the network). (**a**) g = 0.5–b = 0.1. (**b**) g = 1.0–b = 0.4.

In Figure 4, we show the simulation results of the obtained entropy values versus the percentage of corrupted banks for different settings of the default diffusion probability parameters and for different implementations of government’s financial support policies. As we were expecting, the entropy starts to fall during the economic shock, due to the defaults of big banks. Another important feature of this figure is that random governmental support of banks has almost no effect. Meanwhile, support provided to the key nodes of the network—the biggest banks, the “superspreaders”, has a considerable effect which is reflected by the fact that the entropy of the network remains bigger—this policy effectively inhibits the decrease in entropy which is the intrinsic trend during economic shocks. The decrease in entropy is approximately a power law of the cumulative fraction *f* which corresponds to a straight line in these graphs; the exponents—the slopes of the graphs—as shown by dashed lines. The behaviour described above is robust and survives a wide range of the model’s parameters. Figure 4a,b provide two example cases, for b=0.1 and g=0.5 in graph (a), and for b=0.4 and g=1.0 in graph (b). Notice that the effect of targetted governemental support is bigger in case (b). This can be explained by noticing that smaller values of the exponent *b* mean that big banks are more vulnerable with respect to the defaults of smaller partners and, hence, the governmental support is needed more. The graphs (a) and (b) also differ in exponent *g*; however, the bigger value of *g* in case (b) is expected to have an opposite effect: a larger *g* means that larger banks have a smaller probability of a spontaneous default which would mean, the other way around, that support would not be needed. This cannot be seen from the difference in graphs (a) and (b), which is explained by the fact that during a shock, triggered defaults dominate over spontaneous defaults; hence, the parameters associated with the spontaneous defaults are less important than the parameters associated with the triggered defaults.

This result regarding system entropy has a direct implication on the predictability of bankruptcy diffusion or financial contagions inside the network, as it would imply that the randomness of the system decreases as a self-targeting-attack strategy is active within the network.

**Figure 5:**
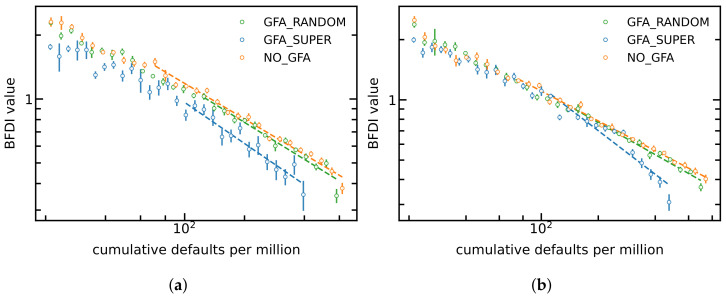
Log–log plot of the simulated BFDI values versus the cumulative number of defaults. (**a**) g = 0.5–b = 0.1. (**b**) g = 1.0–b = 0.4.

In Figure 5, we show in a log–log scale the simulated BFDI values versus the cumulative number of defaults *f*. The observed behaviour is completely in-line what we saw in Figure 4. During an economic shock, the simulated BFDI index decays approximately as a power law of fraction *f*. The curves corresponding to targetted governemental support lie beneath the other curves, evidencing that the applied policy successfully inhibits the propagation of the economic shock. The random governmental support has almost no effect—the corresponding curve almost coincides with the curve with no governemental support.

**Figure 6:**
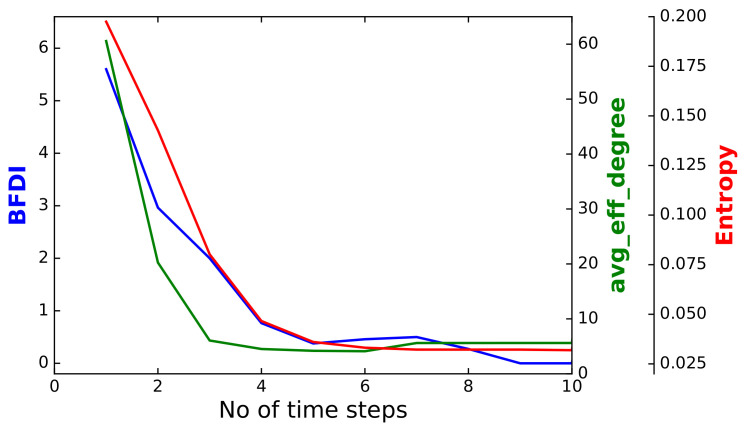
The Bank Failure Diffusion Index, the effective averaged nodes degree and the entropy values obtained from the output of a single simulation run.

The effectiveness of an induced self-targeted-attack strategy is clearly captured in Figure 6, where the BFDI, entropy and the average *effective* degree of the nodes are plotted against time. We use the term *effective* to refer solely to those edges which are, at the given moment in time, connected at both ends to *surviving* banks (nodes). In more detail, the average effective degree is defined as the weighted average degree, with the weights being equal to the squared degree, $k_{\mathrm{avgeff}}=\langle k^{3}\rangle /\langle k^{2}\rangle$, with the angular braces denoting averaging over all the survived nodes of the network: $\langle k^{n}\rangle \equiv \frac{1}{N}\sum_{i}k^{n}$, where $k_{i}$ is the number of the remaining edges of the *i*-th survived node, and *N*—the total number of surviving nodes. The quantity $k_{\mathrm{avgeff}}$ is designed to reflect how the big nodes disappear from the network: the removal of a single big node decreases $k_{\mathrm{avgeff}}$ significantly, while the removal of several small nodes has only a minor effect.

Looking at Figure 6, it appears clear that the initial randomness of the system (entropy) progressively decreases over the same time periods as when the self-targeting attacks break out through the network. Moreover, the effectiveness of a self-targeted strategy is confirmed by the decreasing values of the averaged nodes degree, a result which is in-line with what is expected from the scale-free character of the network (larger banks are expected to default earlier than smaller ones). All this can be seen from the fact that all the three curves move in a strongly correlated way.

Provided that the financial system is characterized by a wide range of interconnections, the analysis conducted in this paper suggests that the financial network has a high sensitivity to the nodes with the highest degree. These nodes can, in various ways, give rise to contagion with potential adverse effects on the system’s stability. An important issue which should be considered when investigating financial-contagion processes is that they can be activated by two channels of interconnectedness: a *direct* one, which concerns all those bilateral links between banks and other financial institutions, and an *indirect* one which relies on common exposures to similar economic sectors due to banks’ common asset holdings, i.e., overlapping portfolios [45]. This latter observation, however, points to a crucial issue: is there an effective way to identify the network’s superspreaders? One way to address this question is to label those banks with the highest Endogenous Risk Index (ERI) or the Indirect Contagion Indicator (ICI), i.e, banks might be ranked by the amount of contagion they could spread in cases of distressed liquidations. The ICI and ERI could allow the quantification of the most important overlaps on a global level and would reveal more valuable information on banks’ interconnectedness than the size of their securities holdings alone [46].

**Table 1:** Scale-free graph and simulations parameters: N: number of nodes in graph; α, β, γ, delta_in, delta_out: see [47] (the above parameters configuration lets us obtain the cumulative degree distribution exponent best matching the observed real data, i.e., k≈2); nr.of runs: number of the simulated samples; max_fails: the maximum number of failures in a single simulation run; max_dur = the maximum number of iterations for a single simulation run; distress_dur = the duration of a bank distress event (equal to one iteration); min_overall_fail_frac: the minimum fraction of failed banks for which the single simulation run is deemed successful.

*NetworkX scale-free parameters*	N = 1000
	α = 0.1
	β = 0.8
	γ = 0.1
	delta_in = 4.5
	delta_out = 4.5
*Simulations parameters*	nr. of runs = 100
	max_fails = 10,000
	max_dur = 60
	distress_dur = 1
	min_overall_fail_frac = 5%

## 6. Concluding Remarks

The high interconnectedness of the global financial system has led to a growing consciousness of the complex dynamics of contagion phenomena. In this study, after having verified the scale-free behaviour of real data from the 2008 financial crisis, we studied if and to what extent the scale-free nature of the network, as being highly vulnerable to self-targeted attacks, can influence the contagion-spreading rate. To this end, by combining a scale-free network approach with Monte-Carlo simulations, we examined how simulated bankruptcy contagion effects spread through the banking system.

The so-far obtained results convey a clear economic interpretation which may provide some insight into how to tackle a contagion at its earliest outbreak. In particular, we show that the initial randomness of the system vanishes as self-targeting attacks spread throughout the network: even though, initially, random network nodes (banks) were infected, very soon the contagion reached the biggest nodes, as evidenced by the steady decrease in both the network entropy, and the Bank Failures Diffusion Index. This has several practical implications for identifying the optimal strategy of averting global financial shocks.

In closing, we would highlight that the results herein presented are very preliminary insights into the topic of modelling economic shocks as a dynamic process over complex networks. There is a clear need for further validation of the simulation results against larger cross-country and cross-sector datasets. In addition, in order to move towards practical applications of our findings for implementing effective financial support policies, further simulations are needed to play out different strategies of averting and suppressing the propagation of financial shocks.

## Data Availability

All empirical data is from The Federal Deposit Insurance Corporation (FDIC): Failed Bank List at https://www.fdic.gov/resources/resolutions/bank-failures/failed-bank-list/ (accessed on 2 October 2022).
